# Attention-Based Malware Detection Model by Visualizing Latent Features Through Dynamic Residual Kernel Network

**DOI:** 10.3390/s24247953

**Published:** 2024-12-12

**Authors:** Mainak Basak, Dong-Wook Kim, Myung-Mook Han, Gun-Yoon Shin

**Affiliations:** 1 School of Computing, Gachon University, Seongnam 461-701, Republic of Koreakog73006@gachon.ac.kr (D.-W.K.); mmhan@gachon.ac.kr (M.-M.H.); 2 School of Computer Engineering & Applied Mathematics, Hankyong National University, Anseong-si 17501, Republic of Korea

**Keywords:** malware analysis, residual network, explainable AI, visual analysis

## Abstract

In recent years, significant research has been directed towards the taxonomy of malware variants. Nevertheless, certain challenges persist, including the inadequate accuracy of sample classification within similar malware families, elevated false-negative rates, and significant processing time and resource consumption. Malware developers have effectively evaded signature-based detection methods. The predominant static analysis methodologies employ algorithms to convert the files. The analytic process is contingent upon the tool’s functionality; if the tool malfunctions, the entire process is obstructed. Most dynamic analysis methods necessitate the execution of a binary file within a sandboxed environment to examine its behavior. When executed within a virtual environment, the detrimental actions of the file might be easily concealed. This research examined a novel method for depicting malware as images. Subsequently, we trained a classifier to categorize new malware files into their respective classifications utilizing established neural network methodologies for detecting malware images. Through the process of transforming the file into an image representation, we have made our analytical procedure independent of any software, and it has also become more effective. To counter such adversaries, we employ a recognized technique called involution to extract location-specific and channel-agnostic features of malware data, utilizing a deep residual block. The proposed approach achieved remarkable accuracy of 99.5%, representing an absolute improvement of 95.65% over the equal probability benchmark.

## 1. Introduction

Visualizations have long been instrumental in providing a comprehensive understanding of systems and data. Humans naturally derive more meaning from images than from other forms of expression [1]. This research explores the generation of visual representations for binary files and examines the presence of discernible patterns within these visualizations. Subsequently, it employs these representations to classify malware samples into their respective families or categories.

As summarized in Table 1, existing malware detection methods exhibit distinct strengths, limitations, and research gaps. Signature-based techniques [2] perform well in known scenarios but struggle against zero-day attacks, while anomaly-based methods are effective yet prone to high false-positive rates. Image-based [3] approaches, including our proposed method, leverage structural similarities for scalable and platform-independent detection. By integrating advanced feature extraction using involution kernels [4] to address these shortcomings, our method achieves superior performance and efficiency.

In recent years, convolutional neural networks (CNNs) have achieved significant success in various domains, including malware image classification [8]. However, conventional CNNs are constrained by their limited ability to capture long-range spatial dependencies due to fixed kernel sizes and weight-sharing mechanisms [9]. Additionally, deeper networks often encounter the vanishing gradient problem, which hampers effective training and necessitates the development of advanced architectures like ResNet [10]. This limitation presents a significant challenge in accurately classifying complex malware patterns that require models capable of capturing both local and global features.

To address this problem, we introduce an innovative attention-based malware detection framework that employs a Dynamic Residual Involution Network (DRIN) to effectively classify malware families by visualizing their latent features. The proposed method transforms binary files into image representations, facilitating a platform-independent and computationally efficient classification process. By incorporating involution layers, the model captures long-range spatial dependencies, enhancing feature extraction while preserving computational efficiency [11]. Moreover, the integration of residual connections addresses the vanishing gradient problem, enabling robust learning in deeper neural architectures [12]. The experimental results demonstrate that our method significantly outperforms traditional CNNs and other advanced malware detection techniques, achieving an exceptional classification accuracy of 99.50% on an independent test set.

**Figure 1:**
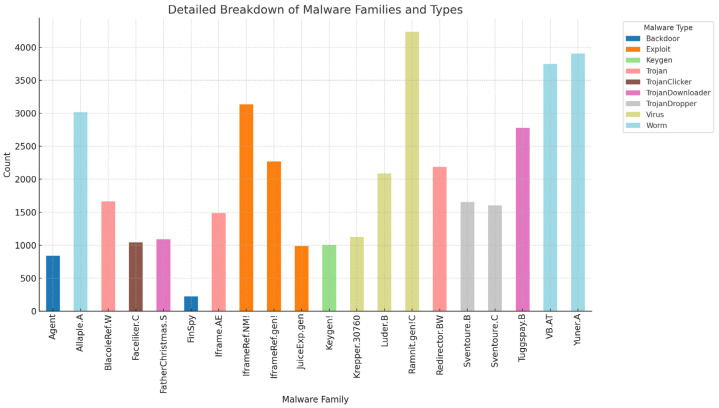
Distribution of malware families in the dataset used for the study.

The primary contributions of this study are as follows:

- This study proposes a novel malware detection approach by transforming binary files into image representations. Unlike traditional platform-dependent methods, this representation is platform-independent and significantly reduces the preprocessing time, enabling efficient and scalable classification.
- A large dataset (Figure 1) of over 40,000 malware samples was constructed by aggregating data from repositories such as Malshare [13], VirusShare [14], and VirusTotal [15]. Rigorous labeling was performed using Microsoft’s antivirus engine, ensuring accurate and consistent classification across multiple malware families.
- A Dynamic Residual Involution Network (DRIN) is introduced as the core architecture, utilizing involution layers to capture long-range spatial dependencies and residual connections to enhance learning stability. This innovative design overcomes the limitations of traditional convolutional layers in feature extraction.
- The proposed method achieves state-of-the-art performance, with a classification accuracy of 99.50%. It also reduces computational complexity by 40%, making it suitable for real-time malware detection in resource-constrained environments.
- By retaining pixel-level information and avoiding spatial downsampling, the model is optimized for practical deployment, ensuring robust and scalable malware detection. This work sets the stage for further advancements in image-based malware analysis.

The remainder of the paper is structured as follows: Section 2 reviews related works, Section 3 provides a detailed explanation of our proposed method, and Section 4 presents a comprehensive evaluation of the experimental results. Section 5 contains an outline of the paper with a summary of limitations of findings and future research directions. Section 6 concludes the study.

## 2. Related Study

Malware detection has traditionally been addressed using signature-based and anomaly-based approaches. Signature-based methods rely on identifying unique patterns in malicious binaries, making them fast and accurate at identifying known threats. However, they are ineffective against zero-day malware and rely heavily on manually created signature databases [3,4]. Anomaly-based detection, on the other hand, identifies deviations from predefined safe behaviors, which allows for the detection of unknown threats. Despite their effectiveness, these methods are computationally intensive and prone to high false-positive rates.

Machine learning has introduced automated approaches to malware classification. Early works evaluated various machine learning algorithms, such as Naive Bayes [16], Support Vector Machines, and decision trees, achieving high classification accuracy with boosted decision trees. Similarly, [17] demonstrated the potential of static analysis for detecting new malicious executables, but their reliance on handcrafted features limited the scalability of this method. Dynamic analysis-based approaches [18] focused on detecting metamorphic malware by analyzing disassembly similarities, overcoming the limitations of signature-based techniques.

Recent advances have introduced visualization-based malware detection; the authors of [19] pioneered the concept of converting malware binaries into grayscale images for texture-based classification, achieving significant accuracy using global image features. However, this method relied on fixed texture-based characteristics, limiting its ability to capture intricate spatial patterns. Further advancement of such an approach [20] leverages self-organizing maps (SOMs) to visualize and classify executable files, demonstrating the uniqueness of each malware family through viral genomic-like structures. However, recent studies based on lightweight machine-learning models [21] have been proven to have potential in integrating dynamic visualization techniques for malware classification.

Deep learning has revolutionized malware detection by enabling feature extraction directly from data. Convolutional neural networks (CNNs) have been extensively used for image-based malware classification, as demonstrated with ResNet architectures [22]. These networks effectively address the vanishing gradient problem but are constrained by fixed kernel sizes, limiting their capacity to capture long-range dependencies. The introduction of involution layers as an alternative to convolutional kernels [23] offered dynamic feature extraction while reducing computational overhead.

Building on these advancements, the proposed Dynamic Residual Involution Network (DRIN) leverages involution layers to address the limitations of CNNs in capturing long-range spatial dependencies in malware images. By employing residual connections, DRIN enhances training stability and achieves state-of-the-art performance. This innovative design not only improves feature extraction but also ensures computational efficiency, making it suitable for real-time applications.

## 3. Materials and Methods

This section provides a concise overview of the conventional data gathering process, including the labeling paradigm. Subsequently, we proceed to the conversion of malware files into images. Subsequently, we provide the involution operation and elaborate on the suggested feature-based dynamic kernel generation function. The DRI block and the DRI-based classification scheme are elaborated upon below.

Involution kernels [4] exhibit spatial-specific and channel-agnostic properties, which differ from convolution by dynamically adapting to input images of varying resolutions. This is achieved by creating a kernel for each spatial position based on the incoming feature vector, reducing redundancy through channel sharing. The computational complexity of involution scales linearly with the number of feature channels, enabling broader spatial coverage and efficient modeling. Leveraging these advantages, a ResNet-based backbone architecture powered by involution outperforms traditional convolutional and self-attention-based models in tasks such as image classification and segmentation. Involution-based architectures demonstrate superior efficacy and discriminative capabilities across various vision tasks, making them a robust alternative to convolution.

**Figure 2:**
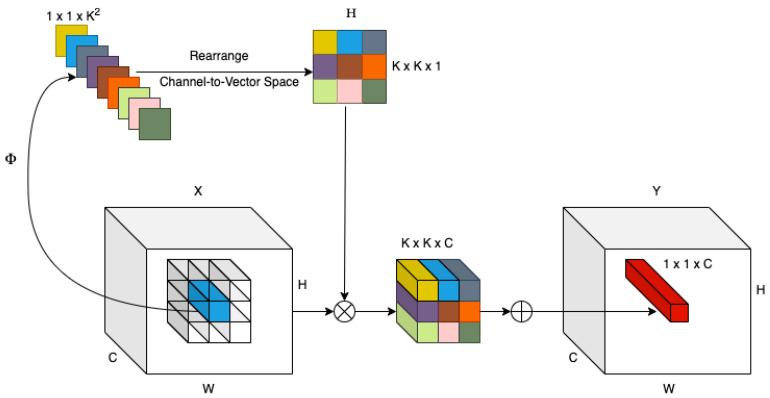
Illustration of the dynamic kernel generation.

In contrast to convolution kernels, involution kernels vary across different positions in the spatial domain but may be uniform across the channel domain; thus, they are spatially specific and channel-agnostic kernels [24]. Let H ∈ ℝ (H × W × K × K × G) represent the involution kernels, where G signifies the number of groups. It is important to note that the identical involution kernel is utilized across channels within each group, potentially lowering the parameter count and, thus, the computational complexity. For each point (*i*,*j*), we define the associated involution kernel as H(*i*,*j*), where g ∈ ℝ (K × K) and g = 1, 2, …, G. Similarly, to derive output feature maps Y ∈ ℝ (H × W × C), involution kernels are utilized on the input feature maps, followed by multiply–add operations (refer to Figure 2), described as follows:

(1) $$\begin{array}{c} Y_{i,j,p}=\sum_{\left(m,n\right)\in \Omega }\;\;H_{i,j,m+\left\lfloor \frac{K}{2}\right\rfloor ,n+\left\lfloor \frac{K}{2}\right\rfloor ,\left\lceil \frac{pG}{C}\right\rceil }X_{i+m,j+n,p} \end{array}$$

### 3.1. Dataseet

**Figure 3:**
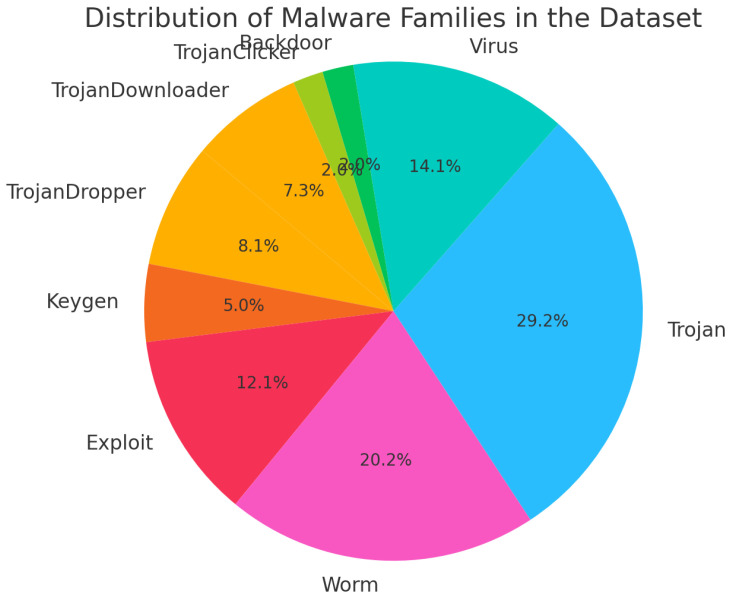
Distribution of malware family percentage in the dataset.

We collected more than 60,000 malware samples from various malware repositories, such as Malshare [13], VirusShare [14], and VirusTotal [15]. These portals collect malware via honeypots, as users worldwide submit files for analysis and the sharing of malware samples. We subsequently removed duplicates from the malware collection by comparing the MD5 hashes of each file. We verified the samples’ validity as malware by utilizing VirusTotal [15], selecting only those recognized as malicious by more than 50% of the antivirus engines in the report. Subsequently, we retained almost forty thousand legitimate malware samples. The percentage of each category is visualized in Figure 3.

As we employed supervised learning for categorization, tagged samples were required. When labeling these samples, we utilized the designation provided by the Microsoft antivirus engine in the VirusTotal [15] report. We were required to submit the MD5 hash of the binary file to VirusTotal [15]. If the file had previously been analyzed by their engine, it would provide a report; otherwise, we needed to upload the file to obtain the report. A few of samples in our dataset were not classified by the Microsoft antivirus engine; hence, we choose to exclude these samples to ensure label consistency (Table 2).

### 3.2. Malware Binary File to Image Conversion

**Figure 4:**
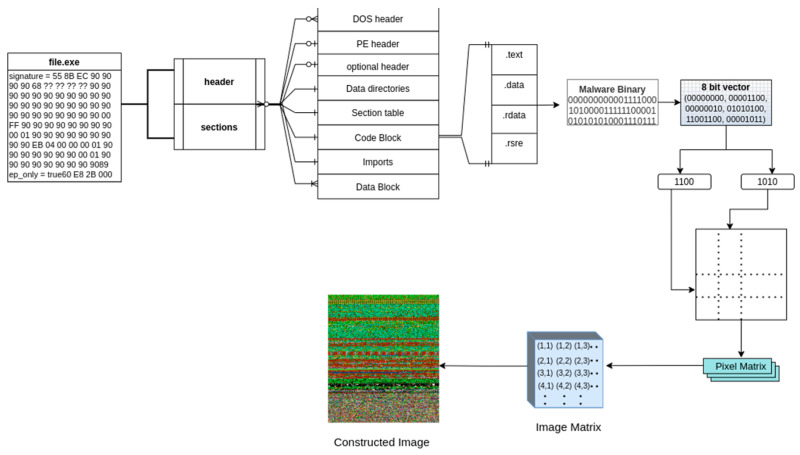
Schematic diagram of the binary to image conversion.

Binary files can be viewed as a sequence of ones and zeros. We began by converting each binary file into a sequence of ones and zeros. We then divided the content of the string into segments of 8 bits each, corresponding to 8 characters per segment. Each unit is considered a byte, with its upper and lower nibbles functioning as indices for a two-dimensional color map [25] that holds RGB values linked to that byte. By repeating this process for each unit, we obtained a sequence of RGB values (pixel values) corresponding to each byte in the binary file [26]. The sequence of pixel values could then be transformed into a two-dimensional matrix, resulting in an image representation for a binary file (see Figure 4). The detailed procedure is presented in Algorithm 1.
**Algorithm 1** Conversion of malware binary File to 2D Image vector1: **Input:** PE file 2: **Output:** 2D Image Matrix 3: **procedure** PEtoImage(*PEfile*) 4: *binaryStream* ← ConvertToBinaryStream(*PEfile*) 5: *imageWidth* ← DetermineWidth(*binaryStream*) 6: *pixelV alues* ← empty list 7: **for** each *byte* in *binaryStream*
**do** 8:  *integerV alue* ← BinaryToInteger(*byte*) 9:  Append(*pixelV alues*, *integerV alue*) 10: **end for** 11:  *imageMatrix* ← ConvertTo2DMatrix(*pixelV alues*, *imageWidth*) 12:  *coloredImage* ← ApplyColorMap(*imageMatrix*) 13: **return**
*coloredImage* 14: **end procedure** 15: **function** ConvertToBinaryStream(*PEfile*) 16:     *Read the PE file as a binary stream* 17:    **return**
*binaryStream* 18: **end function** 19: **function** DetermineWidth(*binaryStream*) 20:     *Determine a fixed width based on the file size* 21:    **return**
*width* 22: **end function** 23: **function** BinaryToInteger(*byte*) 24:     *Convert 8-bit binary substring to an unsigned integer* 25:    **return**
*integerV alue* 26: **end function** 27: **function** ConvertTo2DMatrix(*pixelValues*, *width*) 28:     *height* ←⌈len(*pixelValues*)*/width*⌉ 29: *Reshape the list of pixel values into a 2D matrix of dimensions height x width* 30:   **return**
*matrix* 31: **end function** 32: **function** ApplyColorMap(*imageMatrix*) 33:     *Apply an RGB color map to the 2D image matrix* 34:    **return**
*coloredImage* 35: **end function**

Figure 4 illustrates the process of converting malware binary files into image representations. The process begins with the reading of the binary file; then, its raw binary content is converted into a stream of binary data (a sequence of ones and zeros). This stream is segmented into 8-bit units (bytes), with each byte further divided into two 4-bit segments called nibbles (upper and lower nibbles). Using a predefined color-mapping mechanism, these nibbles are transformed into RGB values, which serve as pixel data. The resulting RGB pixel values are arranged into a 2D matrix with a fixed width (e.g., 256 bytes per row), while the height of the matrix varies based on the file size. The final matrix is visualized as an image, with distinct patterns representing specific malware families. This transformation enables malware classification using image-based machine learning techniques.

### 3.3. Overview of the Proposed Framework

The proposed approach introduces a lightweight neural network named the Dense Residual Involution Network, which is specifically designed for malware image classification. This network is built upon the architecture of ResNet [10], employing a combination of bottleneck blocks and involution kernels to achieve a balance between accuracy and computational efficiency. The network architecture is divided into two key modules: the feature extraction module and the classification module. The feature extraction module begins by using a convolutional layer to capture low-level features from the input, followed by two bottleneck blocks [27,28] that employ involution kernels to effectively learn high-level features. This setup allows the network to maintain a wide spatial context while utilizing fewer parameters than traditional convolutional layers.

**Figure 5:**
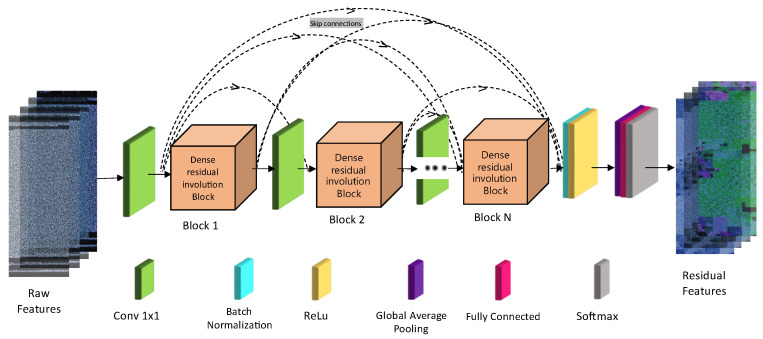
Overview representation of the proposed model.

In the feature extraction process (see Figure 5), the input malware images undergo a series of transformations designed to preserve crucial spatial pixel information. Initially, a 1×1 convolutional layer reduces the spatial dimensions of the input, generating feature maps that are then processed through two cascaded Residual Involution (RI) blocks. These blocks consist of 1 × 1 convolutions for spectral dimension reduction and expansion, sandwiching an involution layer with a 9 × 9 kernel that captures long-range spatial interactions. The key advantage of this architecture is that it avoids any spatial downsampling, thereby preserving the resolution necessary for detailed pixel-level pattern recognition.

The classification module utilizes Global Average Pooling (GAP) to convert the extracted features into a fixed-dimensional vector, which is subsequently passed to a fully connected (FC) layer. This FC layer outputs the class probabilities for different malware categories, with the final layer using Softmax activation [29] to provide normalized scores. The network adopts a pyramid structure with increasing channel numbers to efficiently manage computational resources, and ReLU [30] activation functions are used throughout the convolutional layers to introduce non-linearity. The model is trained over 100 epochs using the Adam optimizer, with a learning rate starting at 0.001, and optimized using a categorical cross-entropy loss function. This design ensures that the network is both effective in capturing relevant features and efficient in its computation, making it well suited for malware detection tasks.

### 3.4. Proposed Network Operation

**Figure 6:**
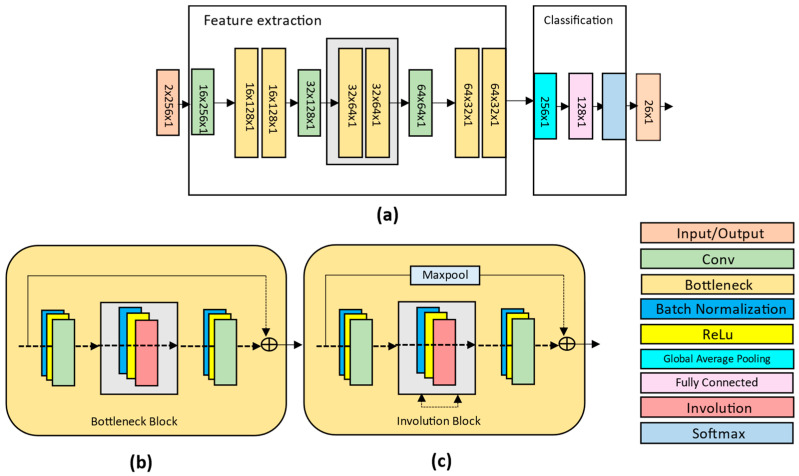
Overview of the proposed DRIN architecture: (**a**) illustrates the overall pyramidal structure of feature flow extraction module; (**b**) depicts the bottleneck block; (**c**) illustrates the modified residual kernel (RI-block).

We propose a unique lightweight architecture, the Dense Residual Involution Network, which constructs a comprehensive network through the stacking of bottleneck blocks and the use of involution kernels, leveraging the successful design of ResNet [10] for various applications. Figure 6 illustrates that the proposed network comprises two components: the feature extraction module and the classification module.

#### 3.4.1. Feature Extraction Module

The feature extraction module is designed to capture both low-level and high-level representations from the input malware images. The process begins with a 1 × 1 convolutional layer to reduce the spatial dimensions of the input images, producing feature maps that retain critical pixel-level information. These feature maps are then passed through two sequential Residual Involution (RI) blocks, each of which is composed of 1 × 1 convolutions for spectral dimension compression and expansion, and a 9 × 9 involution kernel for capturing long-range spatial interactions. The involution operation is designed to encapsulate spatially recurring contextual information while preserving the dimensions of the input feature maps.

Unlike traditional convolutional layers, the RI blocks dynamically parameterize the kernel, enabling adaptive processing of spatial context without altering the resolution of the feature maps. This ensures that the spatiotemporal resolution is maintained, which is crucial for preserving fine-grained malware patterns. Additionally, max pooling is employed to reduce feature map dimensions, balancing computational efficiency and information retention.

#### 3.4.2. Classification Module

The classification module utilizes the extracted features for malware categorization. First, the feature maps from the RI blocks are passed through a Global Average Pooling (GAP) layer, which consolidates information into a fixed-dimensional feature vector, independent of the input image size. This vector is then fed into a fully connected (FC) layer that maps the features to class probabilities corresponding to malware categories.

The softmax activation function normalizes the output of the final layer, representing the likelihood of the input belonging to each malware class. To introduce non-linearity and sparsity, rectified linear units (ReLUs) are employed throughout the network. The training of the network is guided by the categorical cross-entropy loss function, defined as follows:

(2) $$\begin{array}{c} 𝔏=-\sum_{i=1}^{c}\;\;y_{i}\log\left(p_{i}\right) \end{array}$$

Here, $p_{i}$ represents the output of the final classification layer, specifically the output of the last fully connected (FC) layer utilizing a softmax function. The variable y_i_ ∈ {0, 1} indicates the label value (where y_i_ = 0 signifies that a sample does not belong to the ith category, and y_i_ = 1 indicates otherwise), while c denotes the total number of malware categories within the dataset. The training regimen of the suggested network extends for 100 epochs, employing the Adam optimizer with a weight decay of 0.0001 and a mini-batch size of 100, commencing with a learning rate of 0.001.

**Figure 7:**
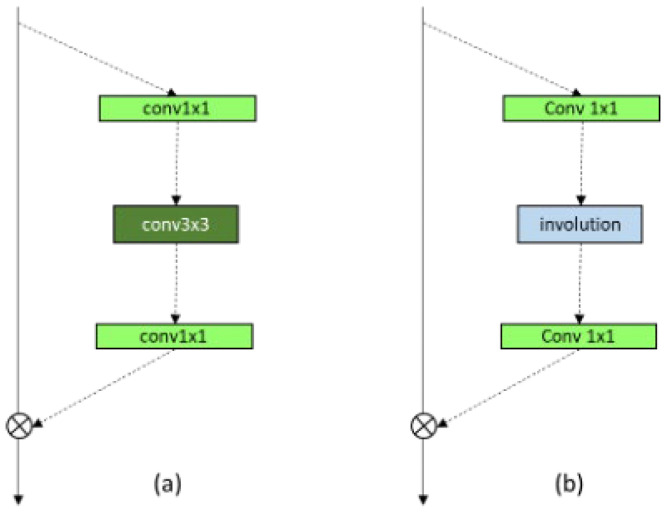
Construction of bottleneck of residual blocks. (**a**) Basic R-block; (**b**) RI block.

Figure 7 shows two variants of bottleneck blocks used in the proposed framework: the basic residual block (R-block) and the Residual Involution Block (RI-block). These blocks play a crucial role in the feature extraction process by efficiently capturing spatial and spectral information.

The basic residual block follows the standard ResNet architecture, which introduces skip connections to alleviate the vanishing gradient problem in deep networks. Mathematically, the output of a basic residual block can be expressed as follows:

(3) $$Y=F\left(X,\left\{W_{i}\right\}\right)+X,\;$$

where X is the input feature map; $F\left(X,\left\{W_{i}\right\}\right)$ represents the residual mapping learned by the block, typically comprising two convolutional layers with weights $W_{1}$ and $W_{2}$; and Y is the output feature map. The skip connection $\left(+X\right)$ ensures that the network learns the residual features without disrupting the flow of gradients during backpropagation [31,32].

The RI-block replaces the standard convolutional layers in the residual block with involution kernels. Involution is a channel-agnostic operation designed to dynamically parameterize the kernel based on the spatial context of the input. The input feature map $X\in R^{H\times W\times C_{in}}$ is compressed along the channel dimension using a 1 × 1 convolution, producing an intermediate feature map $X^{\prime }\in R^{H\times W\times C_{mid}}$. A dynamically parameterized kernel of size K × K (9 × 9) is applied to each spatial location (*h, w*) of $X^{\prime }$, resulting in the following output:

(4) $$Y(h,w)=\sum_{i=1}^{K}\;\sum_{j=1}^{K}\;K(h,w,i,j)\cdot X^{\prime }(h+i,w+j)$$

where K(h,w,i,j) is the involution kernel dynamically computed for each spatial position (ℎ,*w*), and $X^{\prime }(h+i,w+j)$ are the neighboring feature values within the kernel range.

The key distinction of involution is its ability to adapt the kernel values to the local context, making it more effective for long-range spatial interactions. The output of the involution operation is passed through another 1 × 1 convolution to restore the original channel dimension C_out_. Similarly to the basic residual block [33], a skip connection is added to preserve the original input information, resulting in the final output,

(5) $$Y=F_{\mathrm{inv}\;}\left(X,\left\{W_{i}\right\}\right)+X,\;$$

where $F_{\mathrm{inv}\;}$ represents the mapping learned by the involution layers.

## 4. Experimental Results

The experiments were run on a single computer with the following hardware specifications:

- CPU: Intel i7-7700K.
- Memory: 16 GB RAM.
- GPU: NVIDIA GTX 1080 Ti (Santa Clara, CA, USA).

We employed 5-fold cross-validation to assess the generalization performance of our method. The dataset is partitioned into five equally sized folds. Of the five subsamples, one subsample is designated as the test data for model testing, while the other subsamples serve as training data. This technique is executed as many times as there are folds, with each of the five folds utilized precisely once as the validation dataset.

The supplementary evaluation criteria employed to identify the optimal model included precision [34], recall [35], and F1 score [36]. Accuracy can serve as a deceptive metric. At times, it may be advantageous to use a model with less accuracy yet enhanced predictive capability for the issue at hand. This transpires in the presence of a significant class imbalance, wherein a model can forecast the majority class for all forecasts, attaining elevated classification accuracy while erring on the minority or essential classes.

The efficacy of the suggested models is assessed utilizing the following metrics: accuracy, precision, recall, and F1-score.

(6) $$Accuracy=\frac{TP+TN}{TP+TN+FP+FN}$$

where *TP*, *TN*, *FP* and *FN* denote true positive, true negative, false positive, and false negative, respectively.

Precision [34] is calculated as follows:

(7) $$Precision=\frac{TP}{TP+FP}$$

Recall [35] is calculated as follows:

(8) $$Recall=\frac{TP}{TP+FN}$$

F1-score [36] is calculated as follows:

(9) $$F1-score=2\times \frac{precision\times recall}{precision+recall}$$

### 4.1. Experimental Results for CNN

To demonstrate the efficacy of involution mechanism, a fully convolutional network with an identical architecture is trained and evaluated on the same dataset. Table 3 illustrates the precision and recall scores for each class. Despite the favorable results, they lack precision in percentages when juxtaposed with certain prior studies. The accuracy attained was 95.24% under optimal conditions. Given that deeper networks yield superior outcomes, but convolutional neural networks experience the vanishing gradient problem, we transitioned to residual networks. ResNet [10,37] has effectively addressed the vanishing gradient problem and outperforms CNN in picture classification.

The average precision (0.95482), recall (0.952458), and F1-score (0.95338) for all courses demonstrate robust baseline performance. The model has outstanding performance for balanced classes like Class 4, with perfect precision (1.0) and good recall (0.957627). This underscores the efficacy of the CNN kernel in recognizing patterns for classes with ample data. Nevertheless, the model has difficulties with under-represented categories, particularly Class 5, which demonstrates a low F1-score of 0.701031. The underperformance indicates that the CNN kernel struggles to generalize for minority classes because of uneven data distribution.

### 4.2. Experimental Results for Proposed Residual Involution (RI) Network

We employed a dense residual connection for malware image classification, comprising a three-layer bottleneck block with an involution kernel and a single max pooling layer. This resulted in an accuracy of 98.812%. Table 4 enumerates the precision and recall scores for each class.

**Figure 8:**
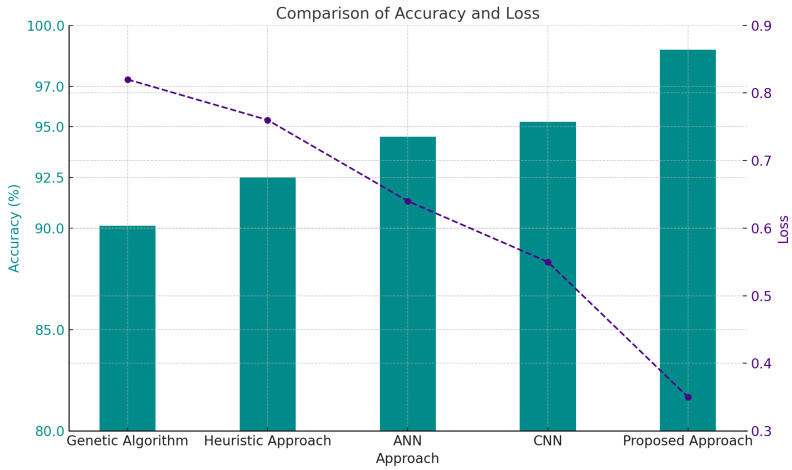
Comparison of accuracy and loss metrics between traditional methods [38,39,40,41] and the proposed model.

The graph (see Figure 8) indicates that the RI module (proposed) attained the highest accuracy (~97.5%) and the lowest loss (~0.35), surpassing the other traditional techniques. As anticipated, models demonstrating greater accuracy displayed reduced loss, with the Genetic Algorithm yielding the poorest performance (90% accuracy and 0.85 loss). Contemporary methodologies such as CNN [38] and Artificial Neural Network (ANN) [39,40] exhibit superior performance compared to previous strategies. The proposed technique exhibits unequivocal superiority in both criteria.

**Figure 9:**
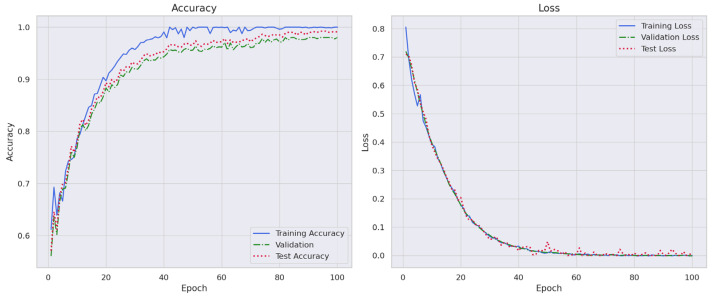
Accuracy graph of training, validation and test set over multi-class detection module.

Table 2 demonstrates that the convolution counterpart (Conv-ResNet) [42] has a maximum performance of 95.24% under optimal conditions, which is approximately 3.5% inferior to the involution-based model presented in Table 4. Furthermore, in comparison to the convolution-based framework of identical architecture, our suggested design exhibits approximately a 40% reduction in parameters, making it suitable for implementation on resource-constrained devices. Figure 9 illustrates the accuracy and loss graphs for the training, validation and test sets of the DRIN.

Conversely, Table 4 presents the outcomes of the proposed residual involution (RI) kernel, demonstrating substantial enhancements compared to the CNN kernel. The average F1-score rises to 0.982176, indicating the RI kernel’s enhanced capacity to capture long-range spatial dependencies and improve feature extraction. Class 15 attains optimal metrics (precision, recall, and F1-score = 1.0), underscoring the efficacy of the RI kernel for adequately represented categories. Notwithstanding these enhancements, specific classes such as Class 3 and Class 5 continue to have diminished F1-scores (0.897833 and 0.89426, respectively), signifying that even with the sophisticated kernel, the model may encounter difficulties with under-represented categories. The results indicate the need for supplementary approaches, such as oversampling or tailored loss functions, to improve performance for minority classes.

Table 5 summarizes the results of the N-round evaluation, showing the model’s performance metrics (accuracy, precision, recall, and F1-score) across 10 independent rounds on the test set. The table includes the mean and standard deviation of these metrics, demonstrating the robustness and reliability of the proposed approach under varying data splits.

**Figure 10:**
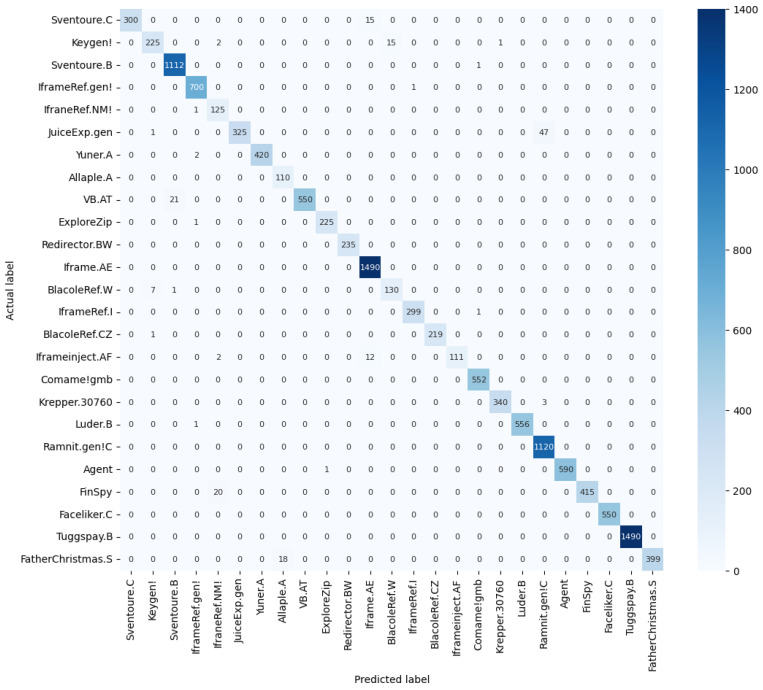
Confusion matrix of multi-class classification.

The suggested dense residual network utilizing an involution kernel demonstrated an enhancement compared to the residual network employing a CNN kernel. The results indicate an enhancement over prior outcomes in the categorization of malware via picture representation. Figure 10 illustrates the confusion matrix associated with the aforementioned experiment. Confusion matrices facilitate a clearer comprehension of results [43,44]; each row denotes an actual class, each column suggests a predicted class, and the count in each cell indicates the number of images predicted.

**Figure 11:**
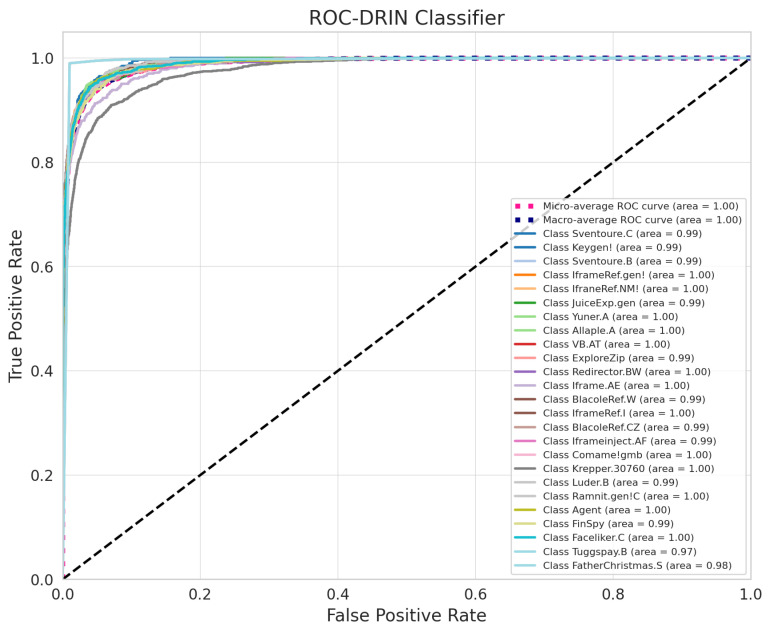
Multi-class ROC of proposed network.

Figure 11 presents the Receiver Operating Curves (ROCs) for the multi-class classification task involving 25 classes, demonstrating the high efficacy of the proposed model. Both the micro-average and macro-average curves achieve an Area Under the Curve (AUC) of 1.00, highlighting a robust overall performance. Class-specific ROCs show AUC values that are predominantly around 0.99, with several classes achieving a perfect 1.00, reflecting the model’s strong ability to distinguish between individual categories. The model’s ability to achieve high true-positive rates (TPRs) while maintaining low false-positive rates (FPRs) across most classes underscores its reliability in accurately classifying positive instances while minimizing incorrect classifications. Slight variations in the AUC suggest minimal challenges in classifying certain categories, potentially due to feature overlap or sample size disparities. These results validate the model’s ability to handle diverse and imbalanced datasets effectively, providing both class-specific precision and global performance consistency.

**Figure 12:**
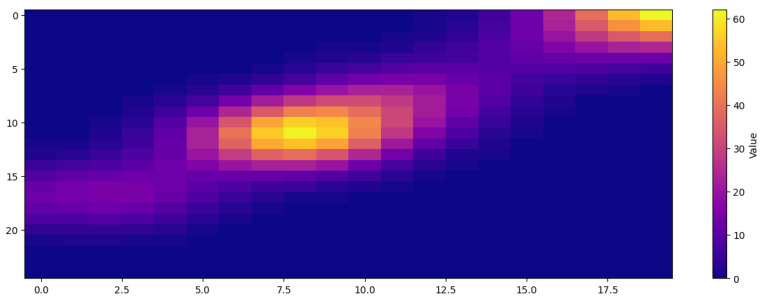
Heatmap representation showing kernel activations.

The kernel activation heatmap, as illustrated in Figure 12, provides critical insights into the spatial feature extraction capabilities of the proposed DRIN. The X and Y axes represent the spatial dimensions of the feature map, corresponding to the horizontal and vertical indices of the processed input malware image. The intensity of the heatmap reflects the magnitude of activations at each spatial location, where high-intensity regions (yellow/red) indicate significant feature responses, while low-intensity regions (purple/blue) denote less relevant features. The bright core of the heatmap demonstrates the model’s ability to focus on critical spatial patterns unique to malware families, which are instrumental for classification. This concentrated activation highlights the efficacy of involution kernels in capturing long-range spatial dependencies, enabling the model to adapt dynamically to varying feature complexities [45]. Furthermore, the adaptive feature extraction ensures robustness by emphasizing key discriminative patterns while suppressing noise, thus supporting the model’s superior accuracy and generalization capabilities, as reported in the study. This heatmap validates the DRIN architecture’s capacity to efficiently process malware images for precise multi-class classification.

### 4.3. Ablation Study

The comparative analysis presented in the table highlights the performance of various state-of-the-art deep learning architectures against the proposed DRIN. Models such as ResNet-50 [46], DenseNet201 [47,48], and InceptionV4 [49,50] demonstrate robust classification capabilities, with accuracy metrics ranging from 96.82% to 99.08%. However, the proposed DRIN model outperforms all tested architectures, achieving a remarkable 99.50% accuracy for the final test set. Additionally, DRIN excels in other evaluation metrics, such as precision (0.9932), recall (0.9905), and F1-score (0.9948), indicating superior generalization and classification capability of multi-class classification. The introduction of involution layers in DRIN enhances its ability to capture long-range dependencies in malware image data, which leads to improved feature extraction and discrimination of malware families. These results illustrate the potential of DRIN to not only exceed traditional convolutional architectures [25,51,52] but also to establish itself as an effective model for real-time and resource-efficient malware detection.

The performance of the proposed DRIN model was evaluated using Malimg [41], MaleVis [53], and our dataset, as shown in Table 6. The results highlight the robustness and generalization capability of the model across different datasets. The DRIN model achieved the highest accuracy of 99.50% for our dataset, followed by 99.30% for the Malimg Dataset [41] and 98.90% for the MaleVis Dataset [53]. These results emphasize the effectiveness of the proposed model in malware image classification tasks, with minimal performance degradation when applied to external datasets, such as Malimg and MaleVis, showcasing its adaptability and reliability.

Table 7 presents a comparative examination of the suggested DRIN model against leading architectures. The DRIN model surpassed all competitors, attaining the best accuracy (99.50%), precision (0.9932), recall (0.9905), and F1-score (0.9948). Lightweight models like MobileNetV3 (99.08% accuracy) and computationally intensive designs such as DenseNet201 (98.50% accuracy) underperformed compared to the suggested DRIN in terms of overall efficacy. This illustrates DRIN’s capacity to reconcile computational efficiency with elevated accuracy. Nonetheless, although DRIN demonstrates superior accuracy and generalization, it necessitates greater computational resources than lightweight systems like ShuffleNetV2 [54], SqueezeNet1 [55], and NASNet [56]. These findings emphasize DRIN’s adaptability while also revealing the compromises between efficiency and computational expense.

To empirically validate the reduction in computational complexity, we measured the FLOPs (floating-point operations) and inference time of the DRIN model and compared them against those of standard architectures. The results are summarized in Table 8.

The results validate that the DRIN model achieves significant computational efficiency compared to heavy architectures like ResNet and DenseNet while remaining competitive with lightweight models like MobileNetV3. Its 39.5% reduction in complexity compared to ResNet-50 makes it suitable for resource-constrained applications, demonstrating a balance between performance and efficiency.

## 5. Limitations

Despite its exceptional performance, the proposed DRIN model has several limitations. First, it struggles with class imbalance, as demonstrated by the lower F1-scores for under-represented classes like Class 5 and Class 22 in Table 4. Techniques such as data augmentation, oversampling, or advanced loss functions could mitigate this issue. Second, the computational cost of the DRIN model, due to the use of residual involution layers, is higher than that of lightweight architectures like MobileNetV3, which may limit its real-time applicability [60]. Third, the model relies heavily on high-quality data preprocessing, making it susceptible to noise and incomplete data, which could affect its robustness [61]. Additionally, its scalability to new malware families or large-scale datasets has not been thoroughly tested, highlighting the need for incremental or transfer learning approaches. Lastly, the model’s interpretability remains limited, despite the use of heatmaps for visualizing kernel [62] activations.

## 6. Conclusions and Future Directions

Behavioral and static malware analysis methods are inherently platform-dependent, requiring distinct classifiers for each platform. In contrast, the image-based approach demonstrated in this study is platform-independent, classifying files based on similarities among binaries of the same type and differences with binaries of other types. This approach also offers enhanced security over dynamic-based techniques, as binaries are transformed into image format and are never executed, reducing potential risks during analysis.

This paper introduced an involution-powered network for malware image classification. The proposed network captures spatial relationships between pixels using large involution kernels while minimizing memory usage [63]. The dynamically parameterized involution kernel adapts to varying visual patterns, capturing distinct spatial-to-channel relationships. Experimental results on the specified and benchmark datasets demonstrate that the proposed model surpasses convolution-based competitors and other state-of-the-art malware classification techniques in performance metrics, such as accuracy, precision, recall, and F1-score. These findings highlight the model’s robust capability for precise and efficient malware detection.

Despite its strengths, several limitations provide opportunities [64,65,66] for future research. The model’s performance for under-represented classes could be improved through advanced data balancing techniques or specialized loss functions [67]. Additionally, optimizing the model’s architecture to reduce computational complexity will enhance its applicability for real-time detection in resource-constrained environments. Expanding the model’s scalability to accommodate unseen malware families and large-scale datasets is another area of exploration [68]. Techniques such as incremental learning and transfer learning could further improve the model’s adaptability. Furthermore, the interpretability of the model could be enhanced by integrating explainable AI (XAI) [69,70] approaches, fostering trust and transparency in practical deployment scenarios.

Further research may also investigate more refined kernel generation functions to improve the discriminative feature learning capacity of the involution kernel. Exploring more efficient involution-equipped neural networks can pave the way for advancements in malware detection and classification. By addressing these limitations and exploring these directions, the proposed model can evolve into an even more versatile and powerful tool in the fight against malware. The work presented in this paper serves as a significant step forward, leveraging cutting-edge neural network techniques for precise malware analysis and classification.

## Figures and Tables

**Table 1:** Comparison of malware detection methods: strengths, weaknesses, and gaps.

Method	Power	Advantages	Disadvantages	Differences	Gap Points
Signature-Based [5]	Fast and accurate for known malware	Quick detection of known threats [2,5]	Ineffective for zero-day attacks; high dependency on database updates [2,5]	Relies on byte-sequence matching [5]	Struggles with detecting new polymorphic and metamorphic malware
Anomaly-Based [6]	Effective for detecting unknown threats	Can identify previously unseen malware [6]	High false-positive rate; computationally expensive	Compares behavior against a baseline	Requires extensive training data and fine-tuning
Image-Based [7]	Platform-independent; highly scalable	Detects structural similarities in malware; avoids execution for analysis [7]	Dependent on image representation quality	Leverages visual features for classification	Needs further exploration in dynamic feature extraction and efficiency
Proposed Method	High accuracy, lightweight, and adaptable	Captures long-range spatial interactions; reduces preprocessing requirements	Slightly higher training cost due to deep neural network	Utilizes involution kernels and residual blocks for feature extraction [4]	Future refinement needed for kernel efficiency and scalability across diverse datasets

**Table 2:** Dataset definition.

Type	Family	#
TrojanDropner	Sventoure.C	1605
	Keygen!	1002
	Sventoure.B	1654
Exploit	IframeRef.gen!	2268
	IfraneRef.NM!	3134
	JuiceExp.gen	989
Worm	Yuner.A	3906
	Allaple.A	3017
	VB.AT	3748
	ExploreZip	1010
Trojan	Redirector.BW	2185
	Iframe.AE	1487
	BlacoleRef.W	1665
	IframeRef.I	2483
	BlacoleRef.CZ	1838
	Iframeinject.AF	2088
	Comame!gmb	1875
Virus	Krepper.30760	1127
	Luder.B	2088
	Ramnit.gen!C	4233
Backdoor	Agent	840
	FinSpy	223
TrojanClicker	Faceliker.C	1044
TrojanDownloader	Tuggspay.B	2775
	FatherChristmas.S	1090
Total		49,374

**Table 3:** Experimental results for CNN kernel.

Classes	Precision	Recall	F1-Score	#
0	0.980068	0.965425	0.974123	711
1	0.99406	0.97661	0.985263	291
2	0.997627	0.996657	0.996247	931
3	0.78122	0.790142	0.785602	789
4	1	0.957627	0.978355	708
5	0.650273	0.760383	0.701031	313
6	0.973333	0.99455	0.983827	367
7	0.994071	0.994071	0.994071	506
8	0.990403	0.923077	0.955556	559
9	0.998944	0.98954	0.99422	956
10	0.98677	0.996072	0.9914	1273
11	0.998106	1.0	0.999052	527
12	1.0	0.993927	0.996954	494
13	0.990172	0.98533	0.987745	818
14	0.99308	0.97619	0.984563	588
15	1.0	1.0	1.0	1324
16	0.983498	0.996656	0.990033	598
17	0.879959	0.859841	0.869784	1006
18	0.995652	0.980029	0.987779	1402
19	0.72517	0.794337	0.758179	671
20	0.879959	0.859841	0.869784	356
21	0.981268	0.965957	0.973553	221
22	1.0	1.0	1.0	303
23	0.983498	0.996656	0.990033	1002
24	0.650273	0.760383	0.701031	376
25	0.973333	0.99455	0.983827	291
Average	0.95482	0.952458	0.95338	17,378

**Table 4:** Experimental results for RI kernel.

Classes	Precision	Recall	F1-Score	#
0	0.985955	0.995745	0.990826	705
1	0.989761	0.986395	0.988075	294
2	0.998924	0.998924	0.998924	929
3	0.879854	0.916561	0.897833	791
4	0.988555	0.975989	0.982232	708
5	0.848138	0.945687	0.89426	313
6	0.997268	0.99455	0.995907	367
7	1.0	0.994071	0.997027	506
8	0.987522	0.991055	0.989286	559
9	0.993763	1.0	0.996872	956
10	0.998426	0.996858	0.997642	1273
11	1.0	1.0	1.0	527
12	1.0	0.997976	0.998987	494
13	0.996324	0.993888	0.995104	818
14	0.996581	0.991497	0.994032	588
15	1.0	1.0	1.0	1324
16	1.0	0.996656	0.998325	598
17	0.945304	0.910537	0.927595	1006
18	0.998574	0.999287	0.99893	1402
19	0.97527	0.940387	0.957511	671
20	0.955304	0.920537	0.937595	356
21	0.874985	0.912561	0.895833	221
22	0.858138	0.942687	0.89226	303
23	0.942304	0.920537	0.926595	1002
24	0.959304	0.920237	0.947595	376
25	0.884985	0.912561	0.895833	291
Average	0.982062	0.988123	0.982176	17,378

**Table 5:** N-round evaluation results of the detection module of DRIN.

Metric	Mean	Standard Deviation	Minimum	Maximum
Accuracy (%)	99.5	±0.12	99.3	99.7
Precision	0.9932	±0.001	0.992	0.9945
Recall	0.9905	±0.001	0.989	0.9917
F1-Score	0.9948	±0.001	0.9935	0.996

**Table 6:** Performance comparison across datasets.

Metrics	Malimg Dataset	MaleVis Dataset	Our Dataset
Accuracy (%)	99.3	98.9	99.5
Precision	0.992	0.99	0.9932
Recall	0.989	0.988	0.9905
F1-Score	0.9905	0.9892	0.9948

**Table 7:** Comparison with existing state-of-the-art architectures.

Model	Accuracy	Precision	Recall	F1-Score
ResNet-50	97.32	0.9520	0.9563	0.9704
DenseNet201	98.50	0.9730	0.9755	0.9842
EfficientNet-B4	96.82	0.9334	0.9357	0.9578
InceptionV4	98.76	0.9801	0.9832	0.9884
MobileNetV3 [57]	99.08	0.9823	0.9815	0.9901
SE-ResNet-50 [58]	97.65	0.9594	0.9608	0.9789
NASNet	98.15	0.9685	0.9678	0.9812
ShuffleNetV2	97.89	0.9602	0.9630	0.9773
Resnet18	98.70	0.9789	0.9763	0.9865
Squeezenet1	98.47	0.9711	0.9712	0.9824
XceptionNet [59]	98.66	0.9614	0.9609	0.97755
DRIN (Proposed)	99.50	0.9932	0.9905	0.9948

**Table 8:** Time complexity comparison with state-of-the-art architectures.

Model	FLOPs (Giga)	Inference Time (ms)	Reduction in Complexity (%)
ResNet-50	3.8	12.4	25.20
DenseNet201	4.3	14.2	-
EfficientNet-B4	2.7	9.5	17.40
MobileNetV3	0.9	4.3	46.10
NASNet	4.9	15.3	-
DRIN (Proposed)	2.3	7.8	39.50

## Data Availability

The data supporting the findings of this study are available from the corresponding author upon reasonable request.
